# Coronavirus disease 2019: What we know?

**DOI:** 10.1002/jmv.25766

**Published:** 2020-03-28

**Authors:** Feng He, Yu Deng, Weina Li

**Affiliations:** ^1^ Department of Thoracic Surgery Tongji Hospital, Tongji Medical College, Huazhong University of Science and Technology Wuhan China; ^2^ Department and Institute of Infectious Disease Tongji Hospital, Tongji Medical College, Huazhong University of Science and Technology Wuhan China

**Keywords:** coronavirus, COVID‐19, SARS‐CoV‐2

## Abstract

In late December 2019, a cluster of unexplained pneumonia cases has been reported in Wuhan, China. A few days later, the causative agent of this mysterious pneumonia was identified as a novel coronavirus. This causative virus has been temporarily named as severe acute respiratory syndrome coronavirus 2 and the relevant infected disease has been named as coronavirus disease 2019 (COVID‐19) by the World Health Organization, respectively. The COVID‐19 epidemic is spreading in China and all over the world now. The purpose of this review is primarily to review the pathogen, clinical features, diagnosis, and treatment of COVID‐19, but also to comment briefly on the epidemiology and pathology based on the current evidence.

## Highlights

- This review is primarily to review the pathogen, clinical features, diagnosis, and treatment of COVID‐19, but also to comment briefly on the epidemiology and pathology based on the current evidence.
- We strongly recommend that the criteria of clinical diagnosed cases based on the symptoms, exposure history, and typical manifestations on chest CT imaging should be used in COVID‐19 affected areas that are in shortage of RT‐PCR testing kits to control the COVID‐19 epidemic.

## 1 INTRODUCTION

In late December 2019, an outbreak of an unknown disease called pneumonia of unknown cause occurred in Wuhan, Hubei province, China.^1^ The outbreak has spread substantial to infect 9720 people in China with 213 deaths and to infect 106 people in 19 other countries up to 31 January 2020 (https://www.who.int/docs/default‐source/coronaviruse/situation‐reports/20200131‐sitrep‐11‐ncov.pdf). A few days later, the causative agent of this mysterious pneumonia was identified as a novel coronavirus (nCoV) by several independent laboratories.^2^, ^3^, ^4^ The causative virus has been temporarily named as severe acute respiratory syndrome coronavirus 2 (SARS‐CoV‐2) and the relevant infected disease has been named as coronavirus disease 2019 (COVID‐19) by the World Health Organization, respectively. According to the daily report of the World Health Organization, the epidemic of SARS‐CoV‐2 so far registered 78 630 cases and 2747 deaths in China, spread to 46 other countries that reported a total of 3664 cases by 27 February 2020 (https://www.who.int/docs/default‐source/coronaviruse/situation‐reports/20200227‐sitrep‐38‐covid‐19.pdf). COVID‐19 epidemic has become a global health threat.

Coronaviruses (CoVs) are a group of highly diverse, enveloped, positive‐sense, and single‐stranded RNA viruses.^5^ They cause several diseases involving respiratory, enteric, hepatic, and neurological systems with vary severity among humans and animals.^5^, ^6^ Human CoV infections have traditionally caused a low percentage of annual respiratory infections. There are HCoV‐OC43, HCoV‐229E, HCoV‐NL63, and HCoV‐HKU1, which cause mild respiratory illness.^5^, ^7^ Over the past 2 decades, two novel CoVs, severe acute respiratory syndrome CoV (SARS‐CoV) and Middle East respiratory syndrome CoV (MERS‐CoV), have emerged and cause severe human diseases.^8^, ^9^ During the epidemic, SARS‐CoV infect more than 8000 people worldwide with nearly 800 fatalities, representing its mortality rate around 10%. Whereas MERS‐CoV infected over 857 official cases and 334 deaths, making its mortality rate approximately 35%.^10^, ^11^, ^12^ So far, SARS‐CoV‐2 is the seventh member of the family of CoVs that infects humans. The main symptoms of COVID‐19 included fever, fatigue, and cough, which are similar to that of SARS‐CoV and MERS‐CoV infected cases. There are some overlapping and discrete aspects of the pathology and pathogenesis of these CoVs which cause severe diseases in humans.^13^

Many literature reported the clinical features, virology, pathology, and radiology of COVID‐19, but the comprehensive review is few. The purpose of this review is primarily to review the pathogen, clinical features, diagnosis, and treatment of COVID‐19, but also to comment briefly on the epidemiology and pathology based on the current evidence.

## 2 THE PATHOGEN

The pathogen that causes COVID‐19 is a nCoV that was first identified in the late January 2020, named SARS‐CoV‐2 (also known as 2019‐nCoV).^2^, ^3^, ^4^

SARS‐CoV‐2 is a novel member of CoVs, which are a large group of highly diverse, enveloped, positive‐sense, and single‐stranded RNA viruses.^5^ Recent research reported that SARS‐CoV‐2 likely originated in bats, based on the similarity of its genetic sequence to that of other CoVs.^14^ The intermediate animal host of SARS‐CoV‐2 between a probable bat reservoir and humans is still unknown.^15^ Although this nCoV has genetic features that are compatible with the family of CoV, nevertheless it has distinct gene sequences that are significantly different from previously sequenced CoVs (Table 1). The analysis of samples from seven SARS‐CoV‐2 infected patients suggested that SARS‐CoV‐2 shares 79.5% sequence identity to SARS‐CoV.^3^ Simplot analysis showed that SARS‐CoV‐2 share 96.2% overall genome sequence identity to RaTG13, which is a short RdRp region from a bat CoV.^3^ Phylogenetic analysis revealed that SARS‐CoV‐2 falls into the subgenus Sarbecovirus of the genus Betacoronavirus and is distinct from SARS‐CoV.^2^, ^4^

**Table 1:** Zoonotic coronaviruses that causes serious disease in human

Coronavirus	Affected host	Intermediate host	Potential reservoir host	Disease	Cell receptor	Reference
SARS‐CoV	Humans	Himalayan palm civet/raccoon	Bat	SARS	ACE2	Li et al^16^
MERS‐CoV	Humans	Dromedary camels	Bat	MERS	DPP4	Wang et al^17^
SARS‐CoV‐2	Humans	NR	NR	COVID‐19	ACE2	Wrap et al^18^

Abbreviations: ACE2, angiotensin‐converting enzyme 2; COVID‐19, coronavirus disease 2019; DPP4, dipeptidyl peptidase 4; MERS‐CoV, Middle East respiratory syndrome‐coronavirus; NR, no report; SARS‐CoV, severe acute respiratory syndrome‐coronavirus.

**Figure 1:**
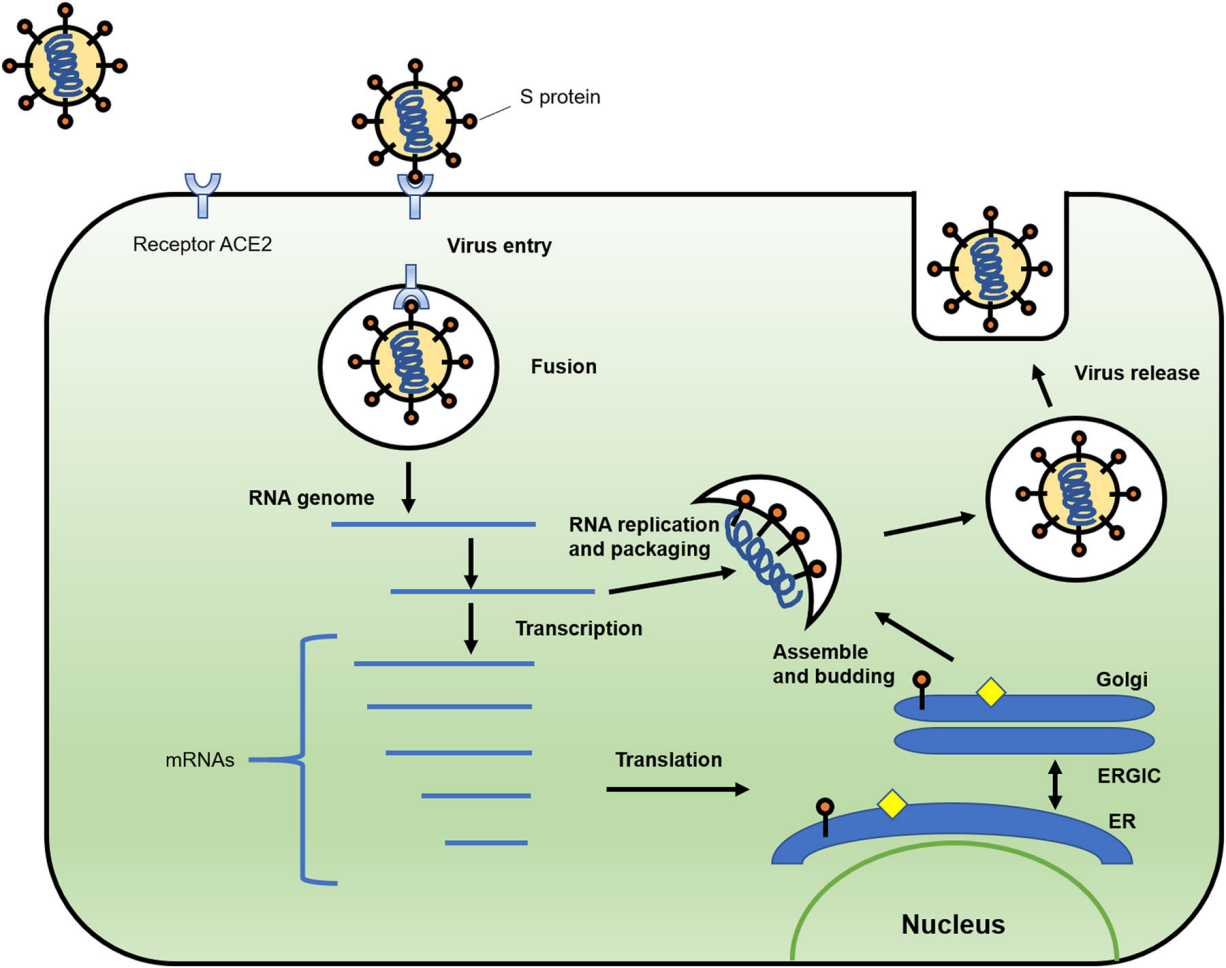
Schematic model of SARS‐CoV‐2 life cycle. S protein binds to the cellular receptor ACE2 to facilitate the entry of the virus. After the fusion of viral and plasma membranes, virus RNA undergoes replication and transcription. The proteins are synthesized. Viral proteins and new RNA genome are subsequently assembled in the ER and Golgi, followed by budding into the lumen of the ERGIC. New virions are released through vesicles. ACE2, angiotensin‐converting enzyme 2; ER, endoplasmic reticulum; ERGIC, endoplasmic reticulum‐Golgi intermediate compartment

The envelope spike (S) protein is important for CoV.^19^ The S protein mediates receptor binding and membrane fusion and is crucial for determining host tropism and transmission capacity.^17^, ^20^, ^21^ Generally, the S protein is functionally divided into the S1 domain, responsible for receptor binding, and S2 domain, responsible for cell membrane fusion.^22^ Structure analysis suggested that receptor‐binding domain was composed of a core and an external subdomain.^19^ Angiotensin‐converting enzyme 2 (ACE2) was known as cell receptor for SARS‐CoV.^16^, ^23^, ^24^ Similar to SARS‐CoV, SARS‐CoV‐2 also use ACE2 as an entry receptor in the ACE2‐expressing cells,^3^ indicating SARS‐CoV‐2 may share the same life cycle with SARS‐CoV (Figure 1).

The biophysical and structural analysis indicated that S protein of SARS‐CoV‐2 binds ACE2 with approximately10‐ to 20‐fold higher affinity than S protein of SARS‐CoV.^18^ The high affinity of S protein for human ACE2 may facilitate the spread of SARS‐CoV‐2 in human populations. Meanwhile, SARS‐CoV‐2 does not use other CoV receptors, such as aminopeptidase N and dipeptidyl peptidase 4 to enter cells.^3^

## 3 EPIDEMIOLOGY

Briefly, cases tend to be in clusters which arrive in waves, and develop into larger outbreaks all over the world. The first documented outbreak occurred primarily in Wuhan.^1^ According to the daily report of the World Health Organization, the epidemic of SARS‐CoV‐2 so far registered 78 630 cases and 2747 deaths in China, spread to 46 other countries that reported a total of 3664 cases by 27 February 2020.

There are evidence suggest that transmission mode is human to human.^25^, ^26^ The major route of transmission of COVID‐19 is droplet and close contact.^26^ Whether infection can occur through the oral or conjunctival routes is unknown, but SARS‐CoV‐2 has been detected in tears,^27^ which is resemble to SARS‐CoV.^28^ Reproductive number (*R*_0_) was estimated by some studies. On the basis of clinical data of patients in COVID‐19 early outbreak, the mean *R*_0_ was ranging from 2.20 to 3.58, meaning that each patient has been spreading infection to two or three other people.^25^, ^29^ It is still too early to develop an accurate *R*_0_ estimate or to assess the dynamics of transmission. More research is needed in the future.

The mean incubation period is about 5 days, ranging from 1 to 14 days and 95% of patients are likely to experience symptoms within 12.5 days of contact.^25^, ^30^ These data suggest a 14‐day medical observation period or quarantine for exposed and close contact persons. However, an asymptomatic carrier was reported and the incubation period was 19 days, suggesting the complicated challenge to contain the outbreak.^31^

## 4 CLINICAL FEATURES

Most case patients were 30 to 79 years of age.^32^ The median age is ranging from 49 to 59 years.^25^, ^26^, ^33^, ^34^ There were few cases in children below 15 years of age. More than half the patients were male. Nearly half the cases had one or more coexisting medical conditions, such as hypertension, diabetes, and cardiovascular disease.^25^, ^26^, ^33^, ^34^ A large cases study indicated that the case‐fatality rate was elevated among those patients with coexisting medical conditions.^32^

The spectrum of clinical presentations of COVID‐19 has been reported ranging from asymptomatic infection to severe respiratory failure.^25^, ^26^, ^30^, ^32^, ^33^, ^34^ The main symptoms include a self‐reported fever, fatigue, dry cough, myalgia, and dyspnea. The uncommon symptoms include sputum production, headache, hemoptysis, and diarrhea.^25^, ^26^, ^30^, ^32^, ^33^, ^34^ Although pneumonia is present in most SARS‐CoV‐2 infected patients, few cases complained of pleuritic chest pain.^26^, ^33^

According to the severity of symptoms, patients can be classified as mild, severe, and critical types^32^ (Table 2). Mild patients had nonpneumonia or mild pneumonia. Severe patients had several clinical findings, including dyspnea, respiratory frequency ≥ 30/min, blood oxygen saturation ≤ 93%, partial pressure of arterial oxygen to fraction of inspired oxygen ratio less than 300, and/or lung infiltrates greater than 50% within 24 to 48 hours. Critical patients had severe conditions, such as respiratory failure, septic shock, and/or multiple organ dysfunction or failure.^32^ If the disease progressed, the median duration period from illness onset to dyspnea was 8.0 days, and to mechanical ventilation was 10.5 days.^34^

**Table 2:** Clinical symptoms associated with COVID‐19

Clinical types	Symptoms
Mild type	Nonpneumonia or mild pneumonia
Severe type	Dyspnea, respiratory frequency ≥ 30/min, blood oxygen saturation ≤ 93%, partial pressure of arterial oxygen to fraction of inspired oxygen ratio < 300, and/or lung infiltrates > 50% within 24/48 h
Critical type	Respiratory failure, septic shock, and/or multiple organ dysfunction or failure

Abbreviation: COVID‐19, coronavirus disease 2019.

Common clinical laboratory findings include leucopenia and lymphopenia.^25^, ^30^, ^33^, ^34^ Lymphopenia is a cardinal feature of COVID‐19. Lactate dehydrogenase and creatinine kinase are all elevated. Half of patients had abnormal liver function, with elevated alanine aminotransferase or aspartate aminotransferase. Most patients had abnormal myocardial zymogram, which showed the elevation of creatine kinase and lactate dehydrogenase. Most patients showed normal serum levels of procalcitonin, but the C‐reactive protein was above the normal range. One‐third of patients had the elevation of D‐dimer.^25^, ^30^, ^33^, ^34^

One study investigated the changes of several cytokines in serum in the COVID‐19 patients.^34^ Initial plasma IL1B, interleukin‐1 receptor antagonist (IL1RA), IL7, IL8, IL9, IL10, basic fibroblast growth factor, granulocyte colony‐stimulating factor (GCSF), granulocyte‐macrophage colony‐stimulating factor, interferon γ, IP10, MCP1, MIP1A, MIP1B, platelet‐derived growth factor, tumor necrosis factor (TNF‐α), and vascular endothelial growth factor concentrations were higher in patients than in healthy adults. Plasma levels of IL5, IL12p70, IL15, eotaxin, and RANTES were similar between patients and healthy adults. Further comparison between intensive care unit (ICU) and non‐ICU patients showed that plasma concentrations of IL2, IL7, IL10, GCSF, IP10, MCP1, MIP1A, and TNF‐α were higher in ICU patients than non‐ICU patients.^34^ These findings suggested that the initiation of the immune response result in the production of chemokines and cytokines, which damage normal host lung.

**Figure 2:**
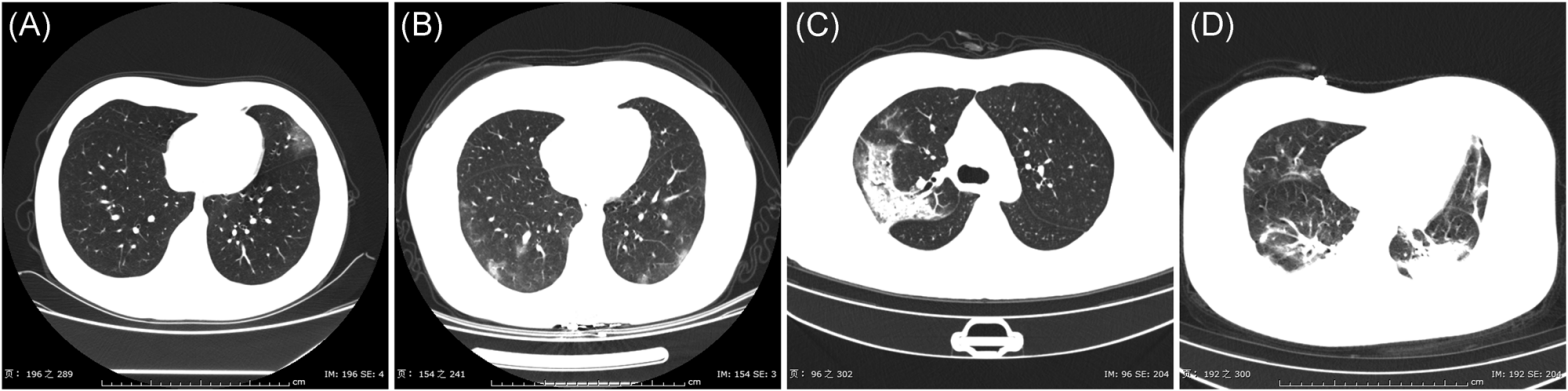
Chest CT Manifestations of COVID‐19. A, Single GGO; B, diffuse GGO; C, consolidation; D, both GGO and consolidation. COVID‐19, coronavirus disease 2019; CT, computed tomography; GGO, ground‐glass opacities

The radiologic manifestations of SARS‐CoV‐2 infected patients are diverse and progressing rapidly.^35^, ^36^, ^37^, ^38^ Two‐third of patients had at least two affected lobes, nearly half of patients had five affected lobes.^37^, ^38^ The most common manifestations are patchy ground‐glass opacities (GGO) and patchy consolidation which were mainly distributed in the middle and outer zone of the lung^37^, ^38^ (Figure 2). A little fibrous stripe may appear if the condition was improved.^37^ One report suggested that there are four stages defined on CT scan.^36^ In early stage, GGO was the main radiological demonstration distributed in the lower lobes unilaterally or bilaterally. In progressive stage, diffuse and bilateral GGO and consolidation in more than two lobes became the main manifestation. In peak stage, the diffuse GGO and dense consolidation became more prevalent. In absorption stage, extensive GGO could be observed and the consolidation was gradually absorbed.

## 5 PATHOLOGY

The pathological findings of human SARS‐CoV‐2 infection have been limited due to the rare number of biopsies or autopsies. In a case reported by Xu et al,^39^ a 50‐year‐old man died 14 days after admission due to respiratory failure and cardiac arrest.^39^ The primary finding of biopsy at autopsy was bilateral diffuse alveolar damage with cellular fibromyxoid exudates and interstitial mononuclear inflammatory infiltrates dominated by lymphocytes. Multinucleated syncytial cells with atypical enlarged pneumocytes characterized by large nuclei, amphophilic granular cytoplasm, and prominent nucleoli were identified in the intraalveolar spaces, showing viral cytopathic‐like changes. No obvious intranuclear or intracytoplasmic viral inclusions were identified. These pathological features show great similarities to SARS‐CoV and MERS‐CoV infection.^40^, ^41^, ^42^ In addition, liver and heart were studied. There is moderate microvascular steatosis and mild lobular and portal activity in the liver tissue and a few interstitial mononuclear inflammatory infiltrates in the heart tissue.

## 6 DIAGNOSIS

Although a good contact history, systemic symptoms, and radiographic changes of pneumonia make the diagnosis likely, the laboratory diagnosis is more reliable. Real time‐polymerase chain reaction (RT‐PCR) is routinely used to detect causative viruses from respiratory secretions.^43^, ^44^ During COVID‐19 transmission events, RT‐PCR has served as the primary clinical laboratory diagnostic test.^25^, ^26^, ^30^, ^33^ Success of these tests are very important to understand the viral kinetics and tissue tropism found in COVID‐19 cases. Several specific and sensitive assays targeting RdRP, N, and E genes of the SARS‐CoV‐2 genome were designed to detect viral RNA in clinical specimens.^44^ Lower respiratory tract samples provide the higher viral loads.^45^ The sampling source or operation may affect RT‐PCR testing results.^43^

The positive rate of RT‐PCR for throat swab samples was reported to be about 60% in early stage of COVID‐19.^46^ These findings suggested that the result of RT‐PCR should be interpret with caution. One study investigated the diagnostic value and consistency of chest computed tomography (CT) compared with RT‐PCR test in 1014 patients with suspected SARS‐CoV‐2 infection. The results suggest that the sensitivity of chest CT in suspected patients was 97% based on positive RT‐PCR result and 75% based on negative RT‐PCR results. These findings indicated that chest CT is a sensitive modality to detect SARS‐CoV‐2 infection.

During the COVID‐19 epidemic in China, 10 567 patients were diagnosed as clinical diagnosed cases. This designation is being used in Hubei province, where is the worst affected area in China. In these cases, no RT‐PCR test was performed but diagnosis was made based on typical symptoms, exposure history, and chest CT manifestations consistent with COVID‐19 pneumonia. Under this criteria, 10 567 cases were diagnosed and isolated. This strategy quarantined a large number of suspected people and protected the healthy people to the most extent. On the basis of experiences above, we strongly recommend that the criteria of clinical diagnosed cases based on the symptoms, exposure history, and typical manifestations on chest CT imaging should be used in COVID‐19 affected areas that are in shortage of RT‐PCR testing kits to control the COVID‐19 epidemic.

## 7 TREATMENT

Until the diagnosis is confirmed, SARS‐CoV‐2 infected patients are treated in single rooms.^25^, ^30^ As SARS‐CoV‐2 is an emerging virus, an effective antiviral treatment has not been identified. The main treatment of COVID‐19 is symptomatic treatment. The antiviral drugs, including oseltamivir, ribavirin, ganciclovir, lopinavir, and ritonavir have been used in attempts to reduce viral load and to prevent the likelihood of respiratory complications in several studies.^25^, ^26^, ^30^, ^33^, ^34^ Remdesivir was reported in the treatment of a patient with COVID‐19 in the United States and got an effective result.^47^ However, the efficacy of these antiviral drugs for COVID‐19 need to be verified by randomized‐controlled clinical trials.

The antibiotics used generally covered common pathogens and some atypical pathogens. When secondary bacterial infection occurred, medication was administered according to the results of bacterial culture and drug sensitivity.^33^ Current evidence in patients with SARS and MERS suggests that receiving corticosteroids did not have a survival benefit, but rather delayed viral clearance.^48^, ^49^, ^50^ Therefore, routine corticosteroids should be avoided unless they are indicated for other reason. Arbidol is used empirically in China because of its direct antiviral effect on SARS‐CoV in cell culture.^51^ Chinese herbal medicine formulae are used to prevent SARS‐CoV‐2 infection in 23 provinces in China.^52^

Noninvasive or mechanical ventilation should be considered in patients with hypoxia despite oxygen supplement and worsening shortness of breath. Extracorporeal membrane oxygenation is used as a last resort.^30^, ^33^, ^34^

## 8 PROGNOSIS

**Figure 3:**
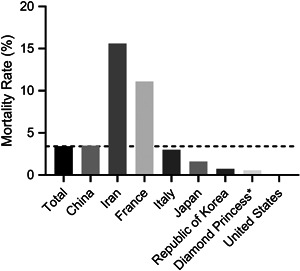
Mortality rates of different countries or regions, 27 February 2020. *A cruise ship currently in Japanese territorial waters

As of 27 February 2020, a total of 2747 deaths in China and 57 deaths outside of China have been reported. The number of laboratory‐confirmed cases and deaths continues to rise. The current reported mortality for COVID‐19 is approximately 3.41% compared to 10% for SARS and 35% for MERS.^10^, ^11^, ^12^, ^53^ The mortality rate was higher than 3.41% in Iran and France, lower in Italy, Japan, Republic of Korea, and United States (Figure 3). Considering the quick spread of COVID‐19, it is still too early to assess the mortality. All countries in the world should respond to the epidemic effectively. Approximately, 20% to 25% SARS‐CoV‐2‐infected patients developed acute respiratory distress syndrome and required ICU care.^30^, ^33^, ^34^ Current evidence indicated that older age and comorbidity may be risk factors for poor outcome.^30^

## 9 SUMMARY AND OUTLOOK

This review summarizes the current findings of SARS‐CoV‐2 along with the treatment for this severe CoV infection. The most common symptoms were addressed. Due to the only biopsy report, the pathological findings associated with SARS‐CoV‐2 infection have been limited. Autopsy is warranted and valuable for future research.

The WHO issued a public health emergency of international concern on 30 January 2020. SARS‐CoV‐2 epidemic is becoming a global concern. At the moment, there is no vaccine and no specific treatment for COVID‐19. The best strategy to deal with SARS‐CoV‐2 epidemic includes controlling the sources of infection, protecting the susceptible people, and cutting off the transmission. The infected patients should be identified early by rapid and robust detection technologies, provided with optimized treatment in isolation timely. The close contact people should be quarantined with follow‐up. The healthy people should be aware of the severity of COVID‐19 and take measures to protect themselves, such as staying at home, limiting social contacts, and wearing protective mask in public. The authorities should encourage people to stay at home; discourage mass gathering; postpone or cancel public events; and close public institutions. These control measures will help COVID‐19 infected countries to prevent the epidemic effectively. Future research will focus on improving the accuracy of early diagnostic tests, developing the vaccine and identifying effective drugs. Therefore, elucidating the pathogenesis of SARS‐CoV‐2 infection is imperative for achieving such goals.

## CONFLICT OF INTERESTS

The authors declare that there are no conflict of interests.
